# Clinical Efficacy of Modified Small Incision Thyroidectomy and Analysis of Influencing Factors of Postoperative Hypocalcemia

**DOI:** 10.3389/fsurg.2022.905920

**Published:** 2022-05-27

**Authors:** Jian Zhou, Hongqing Ju, Hongyan Ma, Qixian Diao

**Affiliations:** ^1^ Second Department of General Surgery, Qingdao Hospital of Traditional Chinese Medicine (Qingdao Haici Hospital), Qingdao, China; ^2^ Operating Room of Qingdao Traditional Chinese Medicine Hospital (Qingdao Haici Hospital), Qingdao, China

**Keywords:** thyroid cancer, modified small incision, efficacy, hypocalcemia, risk factor

## Abstract

**Objective:**

Analyze the clinical effect of modified small incision thyroidectomy and evaluate the influencing factors of hypocalcemia (EH) in patients after operation.

**Methods:**

A total of 220 patients with thyroid cancer in our hospital from October 2019 to October 2021 were selected. The patients were randomly divided into a control group and an observation group, with 110 patients in each group. The control group were treated with traditional thyroidectomy, while the observation group were treated with modified small incision surgery. The perioperative indicators of the two groups were compared. The thyroid hormone indexes of the two groups were meansured before operation and 7 days after operation, and the incidence of complications was compared between the two groups. Serum calcium was detected 7 days after operation in both groups. According to the level of blood calcium, patients were divided into EH group and normal group. The data of two groups were compared, and the related factors affecting the occurrence of EH after operation were analyzed.

**Results:**

The operation time, incision length and intraoperative bleeding volume of patients in the observation group were significantly lower than those of patients in the control group (*p *< 0.05). There was no significant difference in drainage time and postoperative drainage volume between the two groups (*p *> 0.05). The postoperative PTH level of patients in the observation group was significantly higher than that in the control group (*p *< 0.05), but there was no significant difference in FT3, FT4 and TSH levels (*p *> 0.05). The incidence of postoperative complications in the observation group (11.82%) was significantly lower than that in the control group (34.55%). Logistic regression analysis showed that bilateral lymph node dissection, parathyroidectomy and decreased PTH were the independent risk factors for EH in our patient after operation (*p *< 0.05).

**Conclusion:**

The modified small incision operation can effectively reduce the occurrence of surgical trauma and related complications. Bilateral lymph node dissection, parathyroidectomy and PTH decrease are the risk factors for postoperative EH in patients with thyroid cancer. Taking corresponding measures to improve the metabolic function of patients during perioperative period will help to reduce the incidence of postoperative EH in patients with thyroid cancer.

## Introduction

Thyroid cancer is a common malignant tumor in the head and neck, and its incidence is increasing year by year in recent years. The local lump caused by thyroid cancer can lead to swallowing dysfunction, and reduce the quality of life of patients, and seriously threaten their life and health (1–3). Thyroidectomy is the main method to treat thyroid cancer. The effectiveness of traditional excision has been widely confirmed in clinic, but it requires a 6–8 cm long incision, which leads to high risk of postoperative complications and obvious scars, thus increasing the physical and mental burden of patients. Surgery can also cause hoarseness, hypocalcemia (EH) and other complications, which is not conducive to the patient’s recovery (4, 5). How to reduce postoperative complications of thyroid cancer is a difficult problem faced by surgeons. With the development of minimally invasive surgery, the improved mini-incision thyroidectomy has increased the technique of endoluminal instruments, reducing the difficulty of operation, and made some progress in the surgical treatment of thyroid cancer. Modified small incision surgery can reduce the incision to about 2 cm, which has the characteristics of low incidence of postoperative complication and minimal trauma (6–8).

Although surgery can bring a certain radical effect, postoperative complications have a negative impact on the recovery of patient’s physical function. EH is a common complication after thyroidectomy. Acupuncture-like numbness or limb twitching occurs locally in the patients’s body, which has a serious impact on the patient’s neurological function, cardiovascular system and bone. In addition, a few patients even suffer from permanent EH, which leads to body function decline and long-term quality of life decrease (9, 10). The ability of doctors’ to predict EH after thyroid surgery is very important for postoperative management. Early detection of high blood pressure risk would eliminate unnecessary laboratory tests. In this study analyzed the clinical effects of modified small incision thyroidectomy in the treatment of thyroid cancer, evaluated the influencing factors of postoperative EH, aimed to finding a suitable surgical treatment method for thyroid cancer, and discussed the correlation between different factors and EH.

## Data and Methods

### General Information

A total of 220 patients with thyroid cancer in our hospital from October 2019 to October 2021 were selected. The patients were randomly divided into a control group and an observation group with 110 patients in each group. The control group were treated with traditional thyroidectomy, while the observation group were treated with modified small incision surgery. In the control group, there were 39 males and 71 females aging (51.93 **± **6.94) years old, with tumor locations on the left side 52 patients and the right side 58 patients. In the observation group, there were 37 males and 73 females, and they were aged (52.08 **± **7.14) years old. The tumor locations were shown in the left side 56 patients and the right side 54 patients. There was no significant difference in general information such as gender, age and tumor location between the two groups (*p *> 0.05), indicating that they were comparable. This study was reviewed and approved by the Ethics Committee of our hospital, and the patients and their families informed consent.

Inclusion criteria: ① All patients met the diagnostic criteria for thyroid cancer, and were diagnosed by CT/pathological biopsy; ② All patients suffered from unilateral involvement, and there was no respiratory and vocal dysfunction; ③ Tumor stage I–III; ④ All patients met the indications of thyroid surgery and had no history of thyroid surgery; ⑤ If there is no serious complication of cardiovascular and cerebrovascular diseases, liver, kidney, lung and other organ and tissue diseases.

Exclusion criteria: ① Metastasis of thyroid carcinoma; ② Patients suffering from serious diseases of the blood system, immune system or other endocrine system; ③ Patients with incomplete clinical data or incomplete postoperative follow-up data; ④ The expected survival time is <6 months.

### Research Methods

Traditional thyroidectomy was used in the control group. The patient was oprated under general anesthesia with endotracheal intubation. The opration was performed in a supine position, with his head tilted back on the operating table. An incision was made through the anterior sternal notch of the neck of the patient. The length of the arc cut is 6–8 cm. The skin, subcutaneous and zonal muscles and other tissues were peeled off. The white line of the neck and the lateral fascia were cut open to fully expose the thyroid to the surgical field. The thyroid suspensory ligament is separated from the upper pole blood vessel, and then the middle vein was ligated to free the lower pole of the gland, which is used to ligate the lower artery and vein after excision. The thyroid gland is separated between the internal and external fascia, and is ligated, stopped bleeding, and removed. According to the situation, clean the peripheral lymph node, and pay attention to protecting of the superior laryngeal nerve and recurrent laryngeal nerve. After successful hemostasis and confirmation of correct operation, the drainage tube was routinely retained and sutured.

The observation group were treated with modified small incision surgery. The patient received general anesthesia with intubation, and the shoulder should be properly raised to expose the operation area. A transverse incision (about 2 cm in length) was made through the sternal notch at the lower edge of the thyroid gland to dissect the skin, platysma and other tissues, and a surgical cavity similar to the conventional thyroidectomy was constructed to ensure that the required parts of the neck surgery were fully exposed with the traction of the retractor. At the same time, the white line of the neck was cut, the inferior polar artery and vein were raised, and the fascia tissue and parathyroid gland below the inferior polar artery and vein were free. During this period, the injuries of cervical venous plexus and muscle group were avoided. The isthmus of the pretracheal space was severed and the thyroid was towed sufficiently away from the suspensory ligament before the middle vein was severed. separate the recurrent laryngeal nerve and move it to the vicinity of the larynx, where the upper pole was ligated. After resection of the upper parathyroid gland, the thyroid gland is pulled out of the incision. After thyroidectomy, thyroidectomy or subtotal thyroidectomy was performed, and lymph node dissection was performed according to the circumstances. After successful hemostasis and confirmation of correct operation, the drainage tube was routinely retained and sutured.

The operation time, intraoperative bleeding volume, incision length, drainage time, and postoperative drainage volume of the two groups were compared.

The thyroid hormone indexes of the two groups were detected before operation and seven days after operation. Fasting venous blood was collected and centrifuged at 3,500 r/min × 10 min. The levels of free triiodothyronine (FT3), free thyroxine (FT4), thyroid-stimulating hormone (TSH) and parathyroid hormone (PTH) were detected by electrochemical luminescence immunoassay using Roche chemiluminescence immunoassay instrument.

The incidence of complications such as recurrent laryngeal nerve injury, hoarseness, dysphagia, and incision infection 14 days after surgery was compared between the two groups.

Blood calcium of patients in the two groups was detected 7 days after surgery. According to the blood calcium levels of patients, they were divided into EH group and normal group. Biochemical EH: serum calcium level <2.15 mmol/L, with or without EH symptoms; EH symptoms occur, and calcium supplementation is required regardless of EH. The data of the two groups were compared and the related factors affecting the postoperative blood calcium levels were analyzed.

### Statistical Methods

SPSS22.0 software was used for processing. Experimental data were normally distributed, and measurement data were expressed as mean standard deviation $(\bar{x}\pm s)$, and enumeration data were expressed as (%). *t-*test analysis was used for pairwise comparison of measurement data between groups. The count data were tested by *χ*^2^ test. Comparison of single factor differences affecting postoperative blood calcium levels was performed using t test or *χ*^2^ test, and multivariate analysis was performed using Logistic model. The test level was *α *=* *0.05, and *p *< 0.05 indicated that the difference was statistically significant.

## Results

### Comparison of Perioperative Conditions Between the Two Groups

**Figure 1:**
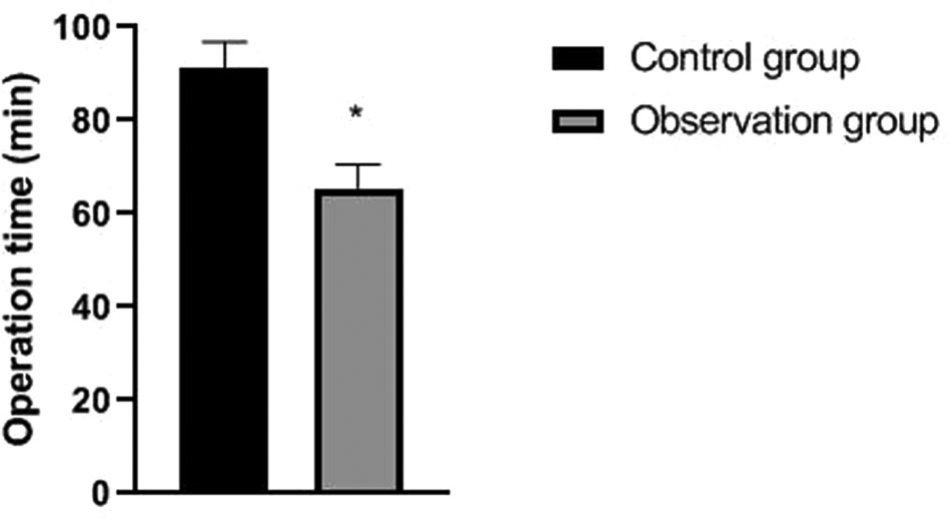
Comparison of operation time between the two groups. Note: compared with the control group, *p < 0.05.

**Figure 2:**
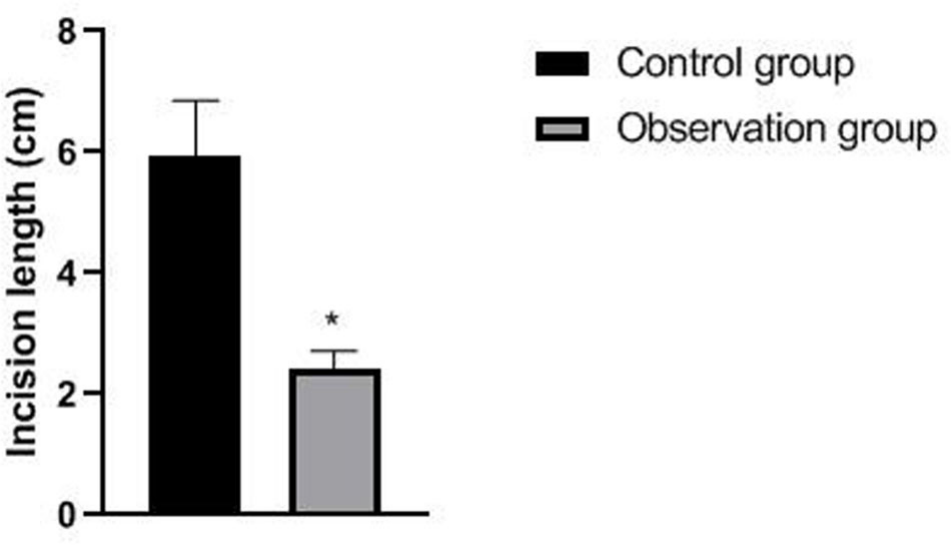
Comparison of incision lengths between the two groups. Note: compared with the control group, *p < 0.05.

**Figure 3:**
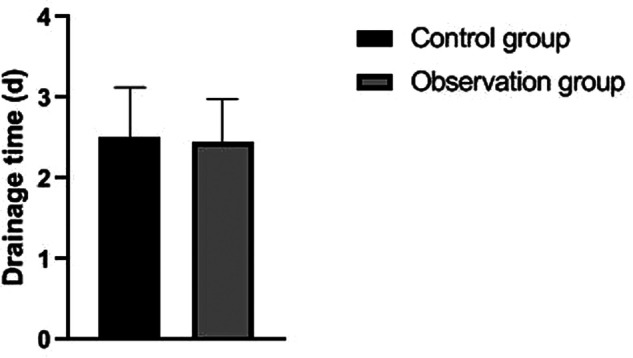
Comparison of drainage time between the two groups.

**Figure 4:**
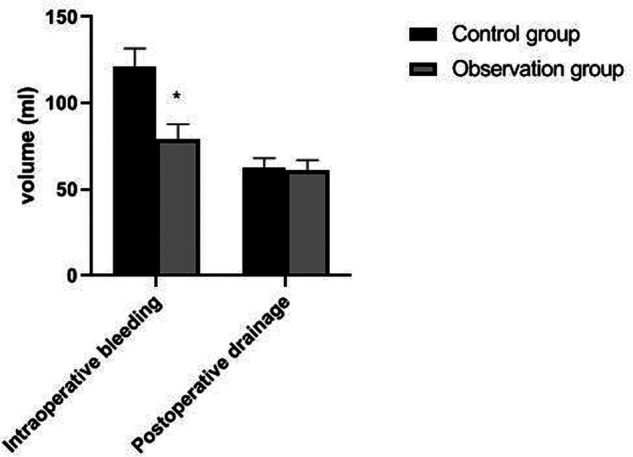
Comparison of intraoperative bleedingv and postoperative drainage between the two groups. Note: compared with the control group, *p < 0.05.

The operation time, incision length and intraoperative bleeding amount of patients in the observation group were significantly lower than those of patients in the control group (*p *< 0.05). There was no significant difference in drainage time and postoperative drainage between the two groups (*p *> 0.05). As showns in Figures 1–4.

### Comparison of Thyroid Hormone Levels Before and After Operation Between the Two Groups

**Figure 5:**
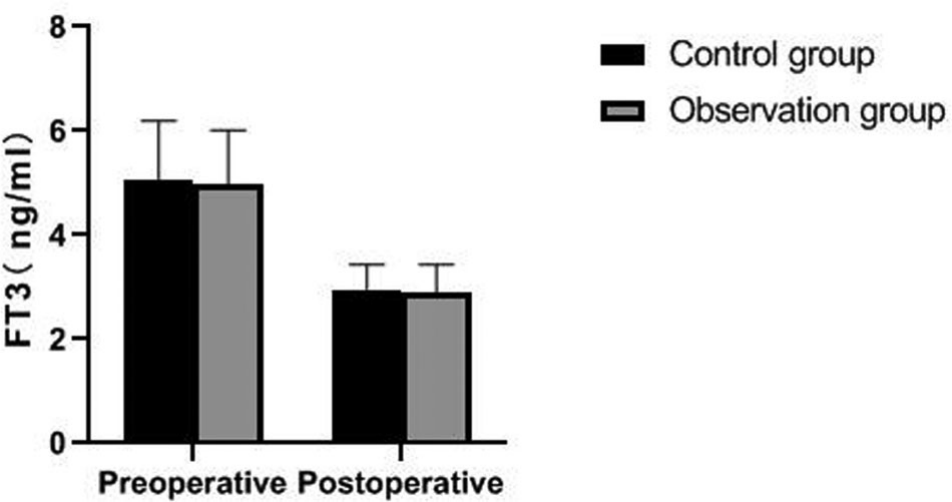
Comparison of FT3 levels between the two groups.

**Figure 6:**
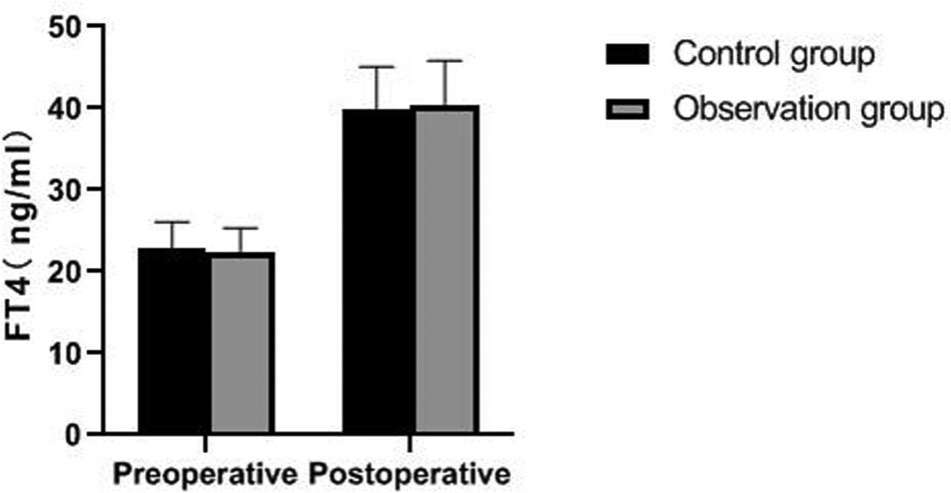
Comparison of FT4 levels between the two groups.

**Figure 7:**
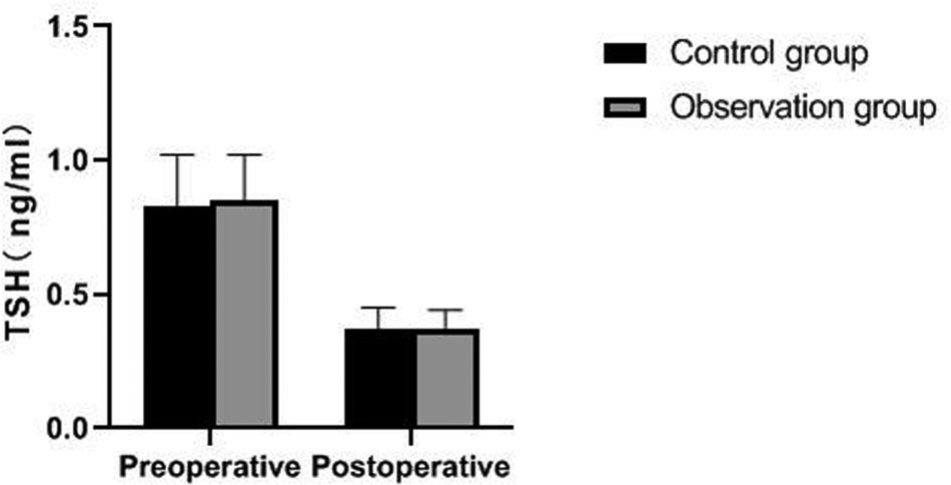
Comparison of TSH levels between the two groups.

**Figure 8:**
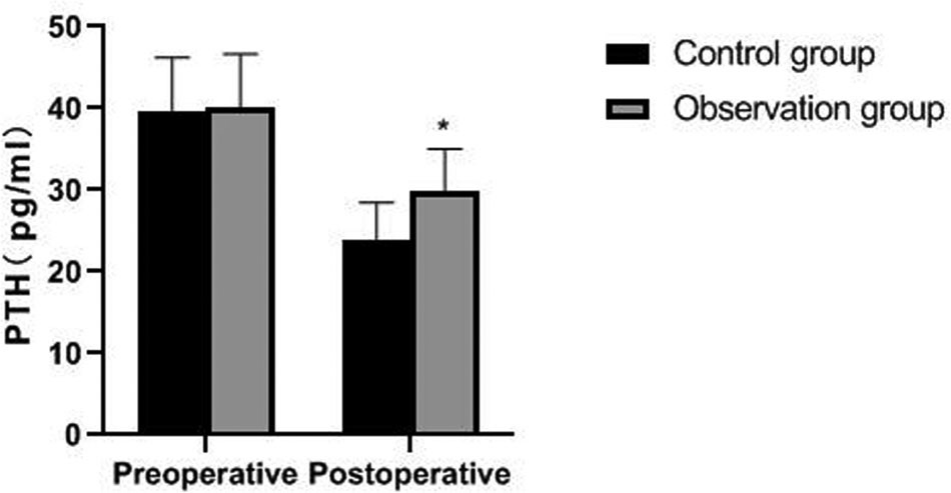
Comparison of PTH levels between the two groups. Note: compared with the control group, *p < 0.05.

There was no significant difference in preoperative FT3, FT4, TSH and PTH levels between the two groups (*p *> 0.05). The postoperative PTH level of patients in the observation group was significantly higher than that in the control group (*p *< 0.05), but there was no significant difference in FT3, FT4 and TSH levels (*p *> 0.05). As showns in Figures 5–8.

### Comparison of the Incidence of Postoperative Complications Between the Two Groups

**Figure 9:**
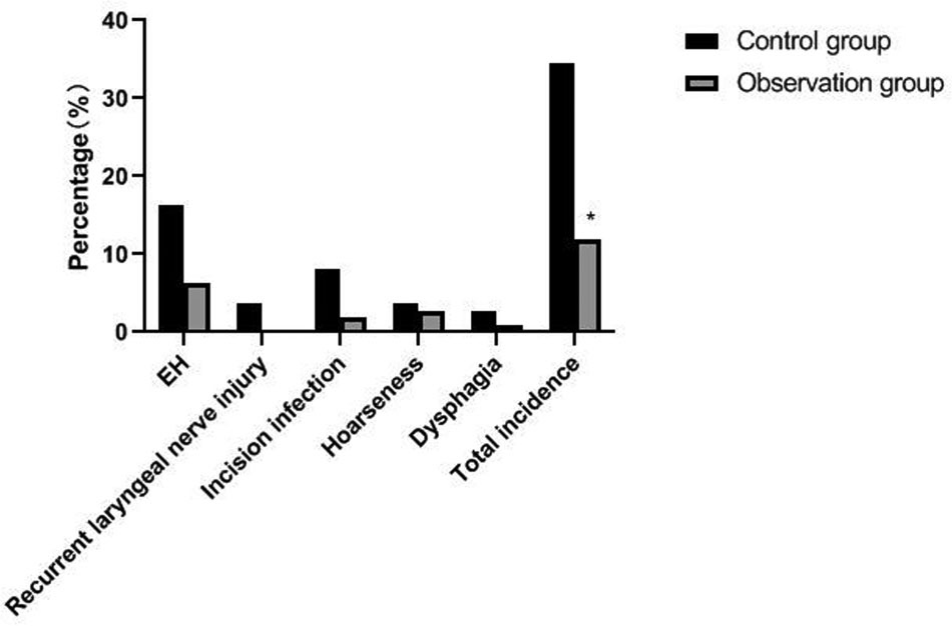
Comparison of incidence of postoperative complications between the two groups. Note: compared with the control group, *p < 0.05.

The incidence of postoperative complications in the observation group (11.82%) was significantly lower than that in the control group (34.55%) (*p *< 0.05). As showns in Figure 9.

### Univariate Analysis of Patients With EH After Surgery

Significant differences were found in the scope of lymph node dissection, parathyroid resection or not, number of cancer foci, operation method, calcitonin and PTH level between the patients with and without EH (*p *< 0.05). As showns in Table 1.

**Table 1:** Univariate analysis of postoperative EH in patients.

Group	Gender		Age (years)		Range of lymph node dissection		Parathyroidectomy	
	male	woman	≥45	<45	unilateral	bilateral	yes	no
EH group (*n* = 25)	9	16	19	six	3	22	14	11
Normal group (*n* = 195)	67	128	174	23	103	92	170	25
*t value*	0.026		2.967		14.789		15.740	
*p value*	0.871		0.085		<0.001		<0.001	
Group	Lymph node metastasis		Number of cancer foci		Surgical approach		Calcitonin (pg/mL)	PTH (pg/mL)
	yes	no	single	multiple	traditional excision	modified small incision		
EH group (*n* = 25)	5	20	10	15	18	7	3.67 ± 0.84	22.25 ± 4.94
Normal group (*n* = 195)	59	136	149	46	92	103	1.49 ± 0.32	27.45 ± 5.58
*t value*	1.130		14.659		5.461		24.977	4.440
*p value*	0.288		<0.001		0.019		<0.001	<0.001

### Multi-Factor Analysis of Postoperative EH in Patients

Logistic regression analysis showed that range of lymph node dissection, parathyroidectomy and decreased PTH were the independent risk factors for EH in our patient after surgery (*p *< 0.05). As showns in Tables 2, 3.

**Table 2:** Assignment for multivariate logistic regression analysis.

Factors	Variables	Assignment
Range of lymph node dissection	X1	Unilateral = 0, Bilateral = 1
Parathyroidectomy	X2	No = 0, yes = 1
Number of cancer foci	X3	Single = 0, Multiple = 1
Surgical approach	X4	Traditional excision = 0, Modified small incision = 1
Calcitonin	X5	Continuous variable
PTH	X6	Continuous variable

**Table 3:** Multivariate analysis of postoperative EH in patients.

Variables	*B*	S.E	Walds	*p*	OR	95% CI
Range of lymph node dissection	0.983	0.378	6.763	0.016	2.672	1.274–5.606
Parathyroidectomy	1.024	0.351	8.511	0.009	2.784	1.399–5.540
Number of cancer foci	0.537	0.279	3.705	0.064	1.711	0.990–2.956
Surgical approach	0.471	0.327	2.075	0.127	1.602	0.843–3.040
Calcitonin	0.453	0.319	2.017	0.268	1.573	0.842–2.939
PTH	1.153	0.327	12.433	0.007	3.168	1.669–6.013

## Discussion

Once thyroid cancer occurs, it can seriously affect the metabolism, nervous and cardiovascular function of the body. Cancerous tissue masses can lead to swallowing and language dysfunction, which poses a great threat to the health of patients (11–13). Surgical thyroid cancer is the most commonly used treatment, especially for high-risk groups. Thyroidectomy can not only prolong the life of patients, but also effectively avoid the oppression and invasion of surrounding tissues by tumors. However, thyroidectomy needs to cut off the anterior cervical muscle and platysma muscle. Severe intraoperative trauma is easy to cause massive bleeding. Incision bleeding and hematoma may cause compression of trachea and esophagus, and even asphyxia.. At the same time, it will damage the recurrent laryngeal nerve and parathyroid gland, resulting in insufficient secretion of parathyroid hormone, lowering of blood calcium content, and even inducing hypocalcemia convulsion, which seriously affecting the postoperative recovery quality (14–17). Therefore, how to carry out effective and low injury resection is an important topic in the treatment of thyroid cancer.

In the past, thyroidectomy was often used in clinical operation, and the injury of recurrent laryngeal nerve was reduced by ligating the upper and lower polar vessels. However, this operation easily damaged the blood supply to parathyroid in patients, which then induced hypoparathyroidism, hypocalcemia and other complications. With the gradual development and improvement of surgical instruments and minimally invasive techniques, as well as the enhancement of people’s health awareness, major changes have taken place in the surgical mode, and various new surgical methods have been continuously promoted. The modified small incision surgery reduced the traditional surgical size from 6–8 cm to about 2 cm, avoided damage to the cervical flap, muscle, blood vessel and nerve, and achieved meticulous thyroid vessel ligation. Resection of the gland lobe while retaining the original position of parathyroid gland has little effect on blood perfusion and thyroid function (18–20). The results of this study showed that the operation time, incision length and intraoperative bleeding volume of patients in the observation group were significantly lower than those of patients in the control group. Compared with the traditional conventional incision group, the modified small incision group has certain advantages in reducing the amount of intraoperative blood loss and the operation time, because it reduces the size of incision and the invasion of surrounding tissues.

Traumatic surgery is easy to cause damage to the surrounding tissues, especially the recurrent laryngeal nerve has no fixed connection with thyroid artery, so it is difficult to determine the specific location of the recurrent laryngeal nerve during the surgical operation. In addition, the distribution of the inferior polar vein vessels in the body is complex and fragile, and bleeding symptoms may occur during the separation process, which will induce various complications (21, 22). Serum PTH is an important index reflecting parathyroid function, while TSH and FT3 are indicators reflecting thyroid function (23, 24). The results of this study showed that the postoperative PTH level of patients in the observation group was significantly higher than that in the control group, but there was no significant difference in FT3, FT4 and TSH levels. From the incidence of postoperative complications in the two groups, the incidence of postoperative EH and recurrent laryngeal nerve injury in the observation group were lower than that in the control group. It indicated that the damage to thyroid caused by the two operations is similar, and the modified small incision operation was more beneficial to protect the parathyroid gland and recurrent laryngeal nerve. Because the operation of a small incision is to pull out the thyroid lobe, then separate, ligate and disconnect it, the difficulty of operation is greatly reduced. Reduce accidental damage to parathyroid gland and recurrent laryngeal nerve.

Calcium plays an important physiological role in the human body, and it involves in nerve transmission, muscle contraction, hormone release and blood coagulation. EH refers to serum total calcium level <2.15 mmol/L or serum free calcium level <1.12 mmol/L (25). The study have pointed out that EH caused by thyroid surgery poses a great threat to patients’ postoperative recovery and quality of life (26). Logistic regression analysis in this study showed that bilateral lymph node dissection, parathyroidectomy and parathyroid hormone lowering were the independent risk factors for EH in our patient after surgery. The extent of lymph node dissection is an independent factor leading to postoperative hypocalcemia. Bilateral lymph node dissection may damage blood vessels and affect the blood supply of parathyroid gland because of its wide range of dissection. Parathyroid cells are very sensitive to the concentration of calcium in the blood. The secretion of PTH increased when the concentration of blood calcium decreased. However, PTH can act on bone cells, increase the permeability of bone cell membrane for calcium ions, and make a large amount of calcium ions in bone cells quickly enter the blood through the bone cell membrane, thus increasing the concentration of calcium ions in the blood (27, 28).

In summary, the modified small incision approach can effectively reduce blood loss, operation time, incision length and other operation-related indexes of patients with thyroid cancer, and reduce the occurrence of surgical trauma and related complications. Bilateral lymph node dissection, parathyroidectomy and the decrease of PTH level are the risk factors for EH in patients with thyroid cancer after operation. It is helpful to reduce the incidence of EH in patients with thyroid cancer after surgery by comprehensive imaging examinations and taking measures to improve the metabolic function of patients.

## Data Availability

The original contributions presented in the study are included in the article/Supplementary Material, further inquiries can be directed to the corresponding author/s.
